# Identification of Two Isoforms of Canine Tetherin in Domestic Dogs and Characterization of Their Antiviral Activity against Canine Influenza Virus

**DOI:** 10.3390/v15020393

**Published:** 2023-01-30

**Authors:** Liang Xu, Jiajun Ou, Xuerui Hu, Yanhong Zheng, Shaotang Ye, Lintao Zhong, Zhiying Lai, Siqi Cai, Gang Lu, Shoujun Li

**Affiliations:** 1 College of Veterinary Medicine, South China Agricultural University, Guangzhou 510642, China; 2 Guangdong Provincial Key Laboratory of Prevention and Control for Severe Clinical Animal Diseases, Guangzhou 510642, China; 3 Guangdong Technological Engineering Research Center for Pet, Guangzhou 510642, China

**Keywords:** tetherin, mutation, isoforms, CIV, restriction

## Abstract

Canine influenza virus (CIV) significantly threatens the canine population and public health. Tetherin, an innate immune factor, plays an important role in the defense against pathogen invasion and has been discovered to restrict the release of various enveloped viruses. Two isoforms of canine tetherin (tetherin-X1 and tetherin-X2) were identified in peripheral blood leukocytes of mixed-breed dogs using reverse transcription polymerase chain reaction (RT–PCR). Amino acid alignment revealed that relative to full-length tetherin (tetherin-X1) and truncated canine tetherin (tetherin-X2) exhibited deletion of 34 amino acids. The deletion occurred at the C-terminus of the coiled-coiled ectodomain and the N-terminus of the glycosylphosphatidylinositol (GPI)-anchor domain. Tetherin-X2 was localized subcellularly at the cell membrane, which was consistent with the localization of tetherin-X1. In addition, canine tetherin-X1 and tetherin-X2 restricted the release of H3N2 CIV. However, canine tetherin-X1 had higher antiviral activity than canine tetherin-X2, indicating that the C-terminus of the coiled-coiled ectodomain and the N-terminus of the GPI-anchor domain of canine tetherin (containing the amino acids deleted in tetherin-X2) are critical for its ability to restrict H3N2 CIV release. This study provides insights for understanding the key functional domains of tetherin that restrict CIV release.

## 1. Introduction

The innate immune response system acts as an important line of defense against pathogen invasion [1]. In animal cells, the sensing of viruses through pattern recognition receptors leads to interferon production and signaling, with the induction of interferon-stimulated genes (ISGs) in hundreds of infected and bystander cells; the proteins encoded by these genes include several classes of autonomously acting proteins (APOBEC3, TRIM5, and tetherin). These proteins are often referred to as “restriction protein” factors [2,3,4].

Tetherin (also named BST2, CD317, and HM1.24), an innate immune factor induced by interferon, also has potential antiviral activity [5,6]. According to previous research, tetherin was discovered to inhibit the release of human immunodeficiency virus type 1 (HIV-1) [7,8]. Tetherin has been shown to restrict the release of various enveloped viruses, such as retroviruses, filoviruses (Ebola and Marburg viruses), arenaviruses (Lassa virus), paramyxoviruses (Nipah and Hendra viruses), and gamma herpesvirus (Kaposi’s sarcoma-associated herpesvirus, KSHV) [9,10,11,12,13,14]. Tetherin contains an N-terminal cytoplasmic tail (CT), a transmembrane domain (TM), a coiled-coiled ectodomain, two potential N-linked glycosylation sites, three extracellular cysteine residues, and a C-terminal glycosylphosphatidylinositol (GPI) anchor [11,12,14,15]. Tetherin is a homodimer with lipid-raft-associated intact type II membrane glycoproteins [16]. The unique membrane topology of tetherin is key to its antiviral activity [17]. The tetherin protein has membrane anchors at its N- and C-termini, which enable its simultaneous insertion into the viral envelope and the plasma membrane [18]. A physical connection is thus formed between the virus and the host cell by tetherin, and this connection impedes the release of progeny virions into the extracellular space [19]. Studies have shown that the TM- and GPI-modified C-terminus of human tetherin drive the insertion of tetherin into the lipid envelope of HIV-I to restrict viral release. The ectodomain is also glycosylated by two N-linked carbohydrate chains that are heterogeneously modified (possibly by polylactosaminoglycan) that cause tetherin to migrate in SDS–PAGE as a smear of 28–45 kDa [15].

Canine influenza virus (CIV) is one cause of canine influenza (CI) in canine populations [20]. CIV was first reported in the United States in 2004 and is currently circulating worldwide [21]. Avian-derived H3N2 CIV was first isolated in Guangdong, China, in 2006 and subsequently reported in dogs in Republic of Korea in 2007; this cross-regional breadth has attracted great attention because it indicates that H3N2 CIV is one of the most prevalent subtypes of CIV [22]. CI poses a serious threat to canine and public health [20].

Tetherin is a lipid-raft-associated apically expressed membrane protein [16]. Influenza viruses use lipid rafts as scaffolds for budding [23]. Studies have shown that tetherin has a significant inhibitory effect on the release of influenza viruses, but the sensitivity of influenza viruses to tetherin is thought to be strain-specific [15]. Canine tetherin (tetherin-X1) has been discovered to mildly restrict CIV release [15]. However, whether the canine genome encodes other isoforms of tetherin and whether these isoforms have antiviral activity against CIV is unknown. In the present study, we amplified a novel isoform of canine tetherin (tetherin-X2). Relative to the previously identified tetherin isoform (tetherin-X1), tetherin-X2 has a deletion of 34 amino acids (aa) at the C-terminus of the coiled-coiled ectodomain and the N-terminus of the GPI anchor. In this study, we investigated the differences between tetherin-X2 and tetherin-X1, the localization of both tetherin isoforms, and the antiviral activity of tetherin-X2.

## 2. Materials and Methods

### 2.1. Viruses and Cells

The H3N2 CIV strain A/canine/Guangdong/02/2014 (GD/2014) was isolated from free-range dogs sick with respiratory diseases in Guangdong Province, China. The virus was propagated in 9- to 11-day-old specific pathogen-free (SPF) embryonated chicken eggs and stored at −80 °C until further use.

Human embryonic kidney (HEK 293T) cells and Madin–Darby canine kidney (MDCK) cells were grown in Dulbecco’s modified Eagle medium (DMEM) (Life Technologies, Carlsbad, CA, USA) supplemented with 10% fetal bovine serum (FBS; Biological Industries, Kibbutz Beit HaEmek, Kibbutz, Israel), 100 U of penicillin, and 100 U of streptomycin (Life Technologies, New York, NY, USA).

### 2.2. Plasmids

To obtain the canine tetherin gene construct, total RNA of peripheral blood leukocytes from healthy mixed-breed dogs was extracted using TRIzol reagent (Takara, Dalian, China). cDNA was synthesized using reverse transcription with a HiScript III 1st Strand cDNA Synthesis Kit (+gDNA wiper) (Vazyme, Nanjing, China). The forward primer (5′-atggcaccgctttaccactactac-3′) and reverse primer (5′-tcaggccagagcagccctaaggccta-3′) specific for canine tetherin (GenBank accession no. XM_038428239) were synthesized based on the NCBI database. The canine tetherin gene was amplified using Phanta Max Super-Fidelity DNA Polymerase (Vazyme, Nanjing, China), and the amplified tetherin gene was inserted into the eukaryotic expression vector pEF-FLAG.

### 2.3. Expression of Canine Tetherin

The full-length pEF-FLAG-Canine-Tetherin-X1 and truncated tetherin pEF-FLAG-Canine-Tetherin-X2 plasmids were transiently transfected into HEK 293T cells. Twenty-four hours post-transfection, a radioimmunoprecipitation assay (RIPA) lysis buffer (Epizyme, Shanghai, China) was used to lyse cells and extract total protein. Western blotting was performed, and total protein was separated via SDS–PAGE and transferred to a PVDF membrane. The PVDF membrane was blocked with 5% (*v*/*v*) skim milk powder diluted in PBS at room temperature for 1 h. After blocking, the PVDF membrane was washed with PBS and incubated overnight at 4 °C with a mouse anti-FLAG monoclonal antibody (Sigma–Aldrich, Burlington, MA, USA) and a rabbit anti-*β*-actin monoclonal antibody (Cell Signaling Technology, Danvers, MA, USA). The PVDF membrane was then incubated with goat anti-mouse IgG H&L (Alexa Fluor^®^ 790) (Abcam, Cambridge, UK) and goat anti-rabbit IgG (Alexa Fluor^®^ 680) (Abcam, Cambridge, UK) at room temperature for 1 h after being washed 3 times with PBS containing 0.1% Tween 20. The PVDF membrane was visualized using an infrared two-color laser imaging system (Odyssey, Lincoln, NE, USA).

### 2.4. Bioinformatics Analysis

The nucleotide and amino acid sequences of the canine tetherin-X1 and tetherin-X2 isoforms were compared with BioEdit software (BioEdit, Borland, Austin, TX, USA). The nucleotide sequences of the tetherin-X1 and tetherin-X2 genes were translated into amino acid sequences using SnapGene (SnapGene, Dotmatics, Bishop’s Stortford, UK) software. The tetherin-X1 and tetherin-X2 amino acid sequences were submitted to I-TASSER (https://zhanglab.ccmb.med.umich.edu/I-TASSER/ (accessed on 20 November 2022)) for 3D simulation of the protein structures.

### 2.5. Immunofluorescence Staining and Confocal Microscopy

HEK 293T cells were transfected with equal amounts of pEF-FLAG, pEF-FLAG-Canine-Tetherin-X1, and pEF-FLAG-Canine-Tetherin-X2 using the transfection reagent Lipo8000 (Beyotime, Shanghai, China). After 24 h of transfection, the culture medium was discarded, and the HEK 293T cells were washed with PBS and fixed with 4% (*v*/*v*) paraformaldehyde at room temperature for 10 min. The 4% (*v*/*v*) paraformaldehyde was discarded, and the cells were then washed with PBS 3 times. QuickBlock™ Blocking Buffer (Beyotime, Shanghai, China) was chosen for blocking at room temperature. The HEK 293T cells were incubated overnight at 4 °C with a mouse anti-FLAG monoclonal antibody (Sigma–Aldrich, Burlington, MA, USA). Then, the HEK 293T cells were washed with PBST containing 0.1% (*v*/*v*) Tween 20 3 times for 5 min each and incubated with fluorescent goat anti-mouse IgG H&L (Alexa Fluor^®^ 488) (Abcam, Cambridge, UK) as the secondary antibody for 1 h at room temperature. Cells were washed with PBST and stained with DAPI (Beyotime, Shanghai, China) for nuclear visualization, and fluorescence was observed using laser confocal microscopy.

### 2.6. Assessment of Whether Canine Tetherin Restricts the Release of CIV

#### 2.6.1. Viral Infection

HEK 293T cells were transfected with pEF-FLAG, pEF-FLAG-Canine-Tetherin-X1, and pEF-FLAG-Canine-Tetherin-X2 separately in 12-well plates. HEK 293T cells were infected with the H3N2 CIV strain A/canine/Guangdong/02/2014 (GD/2014) 24 h after transfection. After incubation with the virus for 1 h at 37 °C in an incubator with 5% CO_2_, the virus solution was discarded, the cells were washed with PBS, and DMEM containing 0.5 µg/mL of TPCK-trypsin, 1% FBS, and 1% penicillin and streptomycin was added. The culture was continued in an incubator with 5% CO_2_ at 37 °C. The virus was collected as described above, and 200 µL of the supernatant was collected at 12 h intervals.

#### 2.6.2. Viral Titer Determination

MDCK cells were harvested and resuspended in DMEM at a concentration of 1.5 × 10^6^ cells/mL. Then, 100 µL of the cell suspension was added to each well of a 96-well plate. The plate was incubated overnight at 37 °C in 5% CO_2_. Serial 10-fold dilutions of the virus were added to each column of wells containing cells. An extra row of mock-infected cells was included across the bottom of the plate as a control. The plates were then incubated for 48 h at 37 °C in 5% CO_2_. The supernatant was discarded after 48 h of infection, and the MDCK cells were washed with PBS. An indirect immunofluorescence assay (IFA) was performed according to the protocol described above. Rabbit polyclonal antibodies against H3N2 CIV NP were prepared and stored at −20 °C. Goat anti-rabbit IgG H&L (Alexa Fluor^®^ 488) (Abcam, Cambridge, UK) was used as the secondary antibody. The fluorescence of the wells was examined under a fluorescence microscope, and the half-maximal tissue culture infectious dose (TCID_50_) was calculated using the Reed–Muench method.

#### 2.6.3. Reverse Transcription Polymerase Chain Reaction (RT–qPCR) Analysis

HEK 293T cells were transfected with pEF-FLAG, pEF-FLAG-Canine-Tetherin-X1, and pEF-FLAG-Canine-Tetherin-X2 separately in 12-well plates. 293T cells, 24 hours after transfection, were infected with the H3N2 CIV strain A/canine/Guangdong/02/2014 (GD/2014) and incubated at 37 °C for 1 h, and the culture medium was then replaced with 1 mL of DMEM containing 0.5 µg/mL of TPCK-trypsin, 1% FBS, and 1% penicillin and streptomycin. The viral supernatant was collected 24 h post-infection, and viral RNA (vRNA) was extracted using the TRIzol method. cDNA was synthesized using a HiScript III 1st Strand cDNA Synthesis Kit (+gDNA wiper) (Vazyme, Nanjing, China), and the reverse transcription primer was designed (IAV-vRNA: 5′-AGTCTTCTAACCGAGGTCGAAACGTA-3′). vRNA was analyzed with RT–qPCR using a ChamQ SYBR qPCR Master Mix (Vazyme, Nanjing, China). Primers (qPCR-CIV-F: 5′-TCAAGTGATCCTCTCGTTATTGC-3′, qPCR-CIV-R: 5′- CACTCTGCTGTTCCTGCCGATA-3′, GAPDH-human-F: 5′- AGATCCCTCCAAAATCAAGTGG-3′, and GAPDH-human-R: 5′-GGCAGAGATGATGACCCTTTT-3′) were synthesized. Similarly, HEK 293T cells were transfected with pEF-FLAG, pEF-FLAG-Canine-Tetherin-X1, and pEF-FLAG-Canine-Tetherin-X2, separately, in 12-well plates. The cells were infected with H3N2 CIV (GD/2014) 24 h after transfection, and total RNA in the cells was extracted 24 h post-infection. Reverse transcription primers (IAV-cRNA: 5′-AACATCCACAGCACTCTGCTGTTCCT-3′, IAV-vRNA, Oligo (dT)) were synthesized for the amplifications of CIV-complementary RNA (cRNA), vRNA, and mRNA. The levels of CIV cRNA, vRNA, and mRNA in infected cells were measured with RT–qPCR using a ChamQ SYBR qPCR Master Mix (Vazyme, Nanjing, China). Differences in expression were calculated using the 2^−∆∆CT^ method.

#### 2.6.4. Western Blot Analysis

HEK 293T cells were transfected with pEF-FLAG, pEF-FLAG-Canine-Tetherin-X1, and pEF-FLAG-Canine-Tetherin-X2, separately, in 12-well plates. The cells were infected with H3N2 CIV (GD/2014) 24 h after transfection, and total protein was extracted 24 h post-infection. A Western blot analysis was performed as described in Section 2.3.

## 3. Results

### 3.1. Amplification of Two Canine Tetherin Isoforms (Tetherin-X1 and Tetherin-X2)

**Figure 1:**
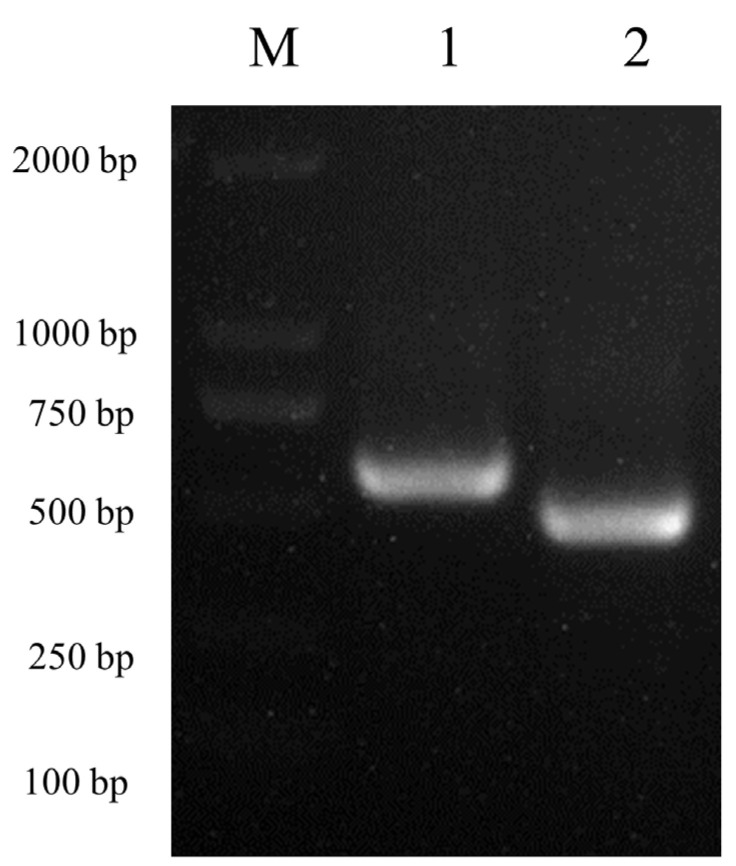
Amplification of two canine tetherin isoforms. Lane M, DL 2000; lane 1, full-length canine tetherin-X1 (567 bp); and lane 2, truncated canine tetherin-X2 (465 bp).

The peripheral blood of mixed-breed dogs was collected, total RNA in the peripheral blood white blood cells was extracted using the TRIzol method, and the canine tetherin gene was amplified using PCR after RT into cDNA. Electrophoresis was carried out on 1% agarose gels at 130 V for 30 min. Based on the sequence of canine tetherin in the NCBI database (GenBank accession no. XM_038428239), the coding region of canine tetherin contains 576 nucleotides and encodes 188 amino acids. The canine tetherin gene (tetherin-X1) amplified from canine peripheral blood cells was consistent with the information in the NCBI database. In addition, a truncated canine tetherin gene (tetherin-X2) was amplified (Figure 1).

**Figure 2:**
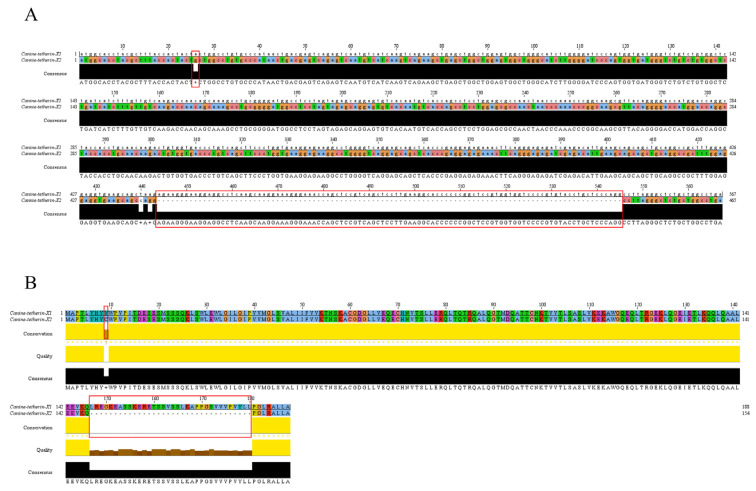
Bioinformatics analysis of the two canine tetherin isoforms (**A**,**B**). Comparison between the nucleotide and amino acid sequences of canine tetherin-X2 and canine tetherin-X1. The nucleotide and amino acid sequences were visualized using Jalview software (https://www.jalview.org (accessed on 2 December 2022)). Red frame indicate lost sequences from Tetherin-X2 alignment to Tetherin-X1.

The truncated canine tetherin gene (tetherin-X2) contained only 465 nucleotides, 102 nucleotides fewer than the full-length tetherin-X1 gene (Figure 2A).

SnapGene software was used to translate the nucleotide sequences of canine tetherin-X1 and tetherin-X2 into amino acid sequences. Relative to full-length tetherin-X1, tetherin-X2 exhibited deletion of aa 147–180 (34 aa), which are located at the C-terminus of the coiled-coiled domain of tetherin and in part of the N-terminal region of the GPI-anchor domain (Figure 2B).

**Figure 3:**
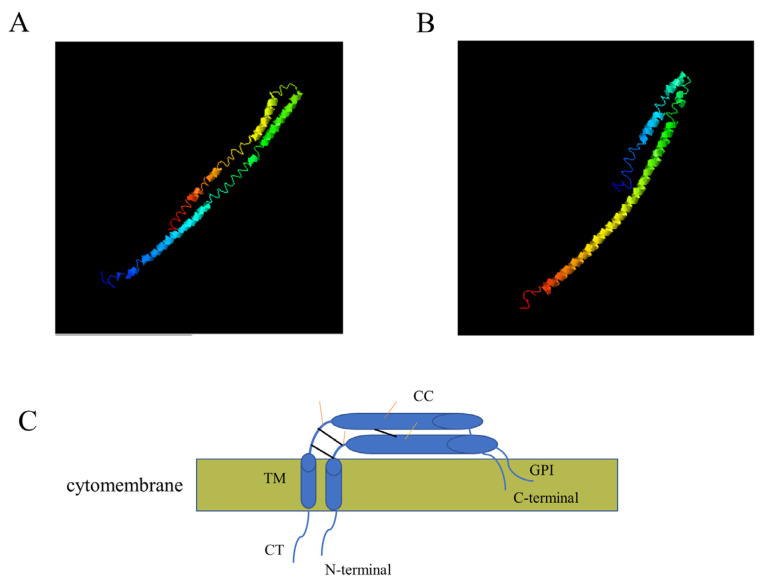
Three-dimensional structures of canine tetherin-X1 and tetherin-X2. (**A**) Simulated 3D structure of canine tetherin-X1; (**B**) simulated 3D structure of truncated tetherin-X2. I-TASSER uses the SPICKER program to analyze all the protein structures in the PDB database and report the five most likely structural cluster models. The feasibility of each model is quantified by the c-score (−5, 2). A higher c-score indicates a greater feasibility of a model. The most reliable models simulating the 3D structures of canine tetherin-X1 and truncated canine tetherin-X2 were chosen. (**C**) The model of the tetherin structure. The black line represents the cysteine site in tetherin. The yellow line represents the glycosylation site in tetherin.

Canine tetherin is a type II transmembrane protein. The 3D structures of canine tetherin-X1 and tetherin-X2 were simulated using I-TASSER software. We chose the most reliable models as the 3D structural models of canine tetherin-X1 (Figure 3A) and canine tetherin-X2 (Figure 3B). The spatial structure of tetherin-X2 was similar to that of tetherin-X1. The amino acid deletion in tetherin-X2 did not alter the spatial structure of the protein.

### 3.2. Expression and Localization of the Two Canine Tetherin Isoforms

**Figure 4:**
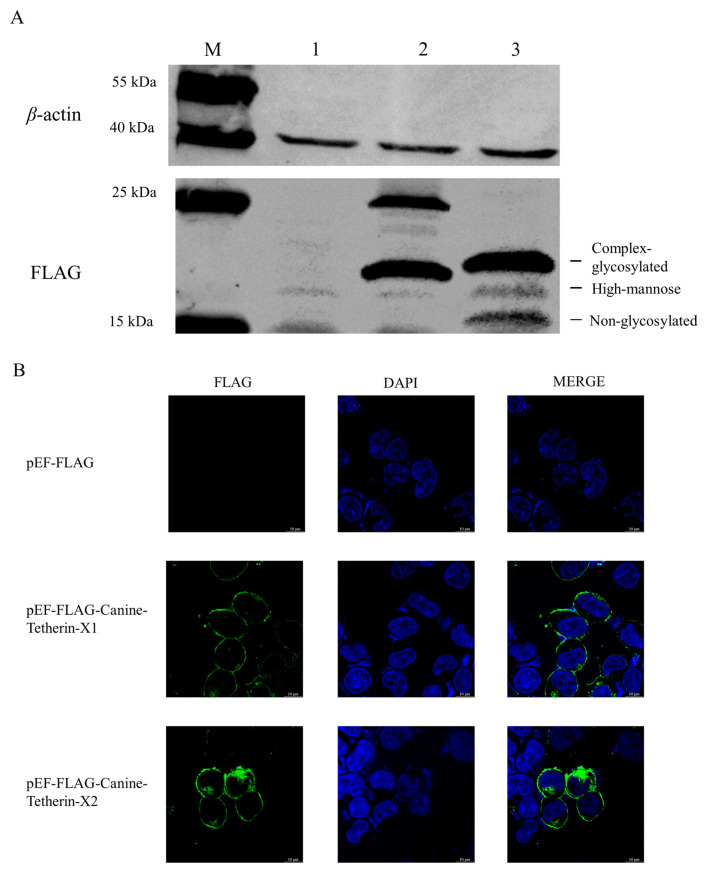
Expression and localization of the two canine tetherin isoforms. (**A**) Expression of the canine tetherin isoforms. HEK 293T cells were transfected with 2500 ng each of the plasmids pEF-FLAG, pEF-FLAG-Canine-Tetherin-X1, and pEF-FLAG-Canine-Tetherin-X2 in a 6-well plate. Twenty-four hours after transfection, the cells were lysed with RIPA buffer. Thirty nanograms of total protein was used for SDS–PAGE. Lane M, protein marker; lane 1, pEF-FLAG; lane 2, pEF-FLAG-Canine-Tetherin-X1; Lane 3, pEF-FLAG-Canine-Tetherin-X2. (**B**) Subcellular localization of the canine tetherin isoforms. HEK 293T cells were transfected with 1000 ng each of the plasmids pEF-FLAG, pEF-FLAG-Canine-Tetherin-X1, and pEF-FLAG-Canine-Tetherin-X2. Twenty-four hours after transfection, IFA was performed. Tetherin is indicated by green fluorescence; nuclei, by blue fluorescence. The experiments were performed in triplicate.

The eukaryotic expression plasmids pEF-FLAG-Canine-Tetherin-X1 and pEF-FLAG-Canine-Tetherin-X2 were transiently transfected into 293T cells. Western blotting and IFA were used to determine the expression and subcellular localization of tetherin-X1 and tetherin-X2. The Western blot results showed that the size of full-length tetherin-X1 was between 15 and 35 kDa and that of truncated tetherin-X2 was between 15 and 31 kDa; in addition, and the Western blot results showed that 3 bands were caused by the migration of tetherin in SDS–PAGE due to its glycosylation pattern (Figure 4A). The subcellular localization of canine tetherin was visualized with laser confocal microscopy. Tetherin-X1 was distributed on the cell membrane, as was truncated tetherin-X2 (Figure 4B).

### 3.3. Canine Tetherin Restricts the Release of CIV

Viral Titer

**Figure 5:**
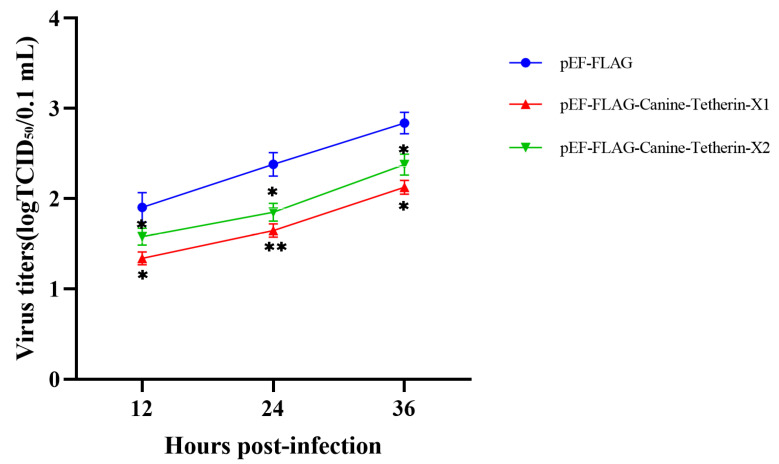
Tetherin restricts the release of H3N2 CIV. HEK 293T cells were transfected with 1000 ng each of the plasmids pEF-FLAG, pEF-FLAG-Canine-Tetherin-X1, and pEF-FLAG-Canine-Tetherin-X2. The cells were infected with H3N2 CIV GD/2014 at an MOI of 0.1 24 h after transfection. The viral titers in the supernatant at each time point were determined using a TCID_50_ assay. MDCK cells were harvested and resuspended in DMEM, and the resuspended cells were seeded in each well of a 96-well plate. The plate was incubated overnight at 37 °C in 5% CO_2_. Serial 10-fold dilutions of the virus were added to each column of wells. An extra row of mock-infected cells was included across the bottom of the plate as a control. The plate was then incubated for 48 h at 37°C in 5% CO_2_. An IFA was performed after 48 h of incubation. The prepared anti-H3N2 CIV NP polyclonal antibody was used. The TCID_50_ was calculated using the Reed–Muench method. Statistical significance was determined using the conventional Student’s *t*-test and calculated with GraphPad Prism software 6. A *p*-value of < 0.05 was considered to indicate significance (* *p* < 0.05; ** *p* < 0.01).

HEK 293T cells were transiently transfected with the eukaryotic expression plasmids pEF-FLAG, pEF-FLAG-Canine-Tetherin-X1, and pEF-FLAG-Canine-Tetherin-X2. Cells were infected with H3N2 CIV GD/2014 24 h after transfection. The viral titer in the supernatant at each time period was measured using a TCID_50_ assay. The viral load in the supernatant from cells transfected with tetherin-X1 was significantly lower than that in the supernatant from control cells at 12 h (*p* < 0.05), 24 h (*p* < 0.01), and 36 h (*p* < 0.05) post-infection (Figure 5). This pattern indicates that canine tetherin-X1 can restrict the release of H3N2 CIV (GD/2014). The viral titer in the supernatant from cells transfected with tetherin-X2 was also significantly lower than that in the supernatant from control cells at 12 h (*p* < 0.05), 24 h (*p* < 0.05), and 36 h (*p* < 0.05) post-infection (Figure 5), indicating that tetherin-X2 can also restrict the release of H3N2 CIV GD/2014. Moreover, at each time point, the viral titers in the supernatant from cells transfected with tetherin-X2 were higher than those in the supernatant from cells transfected with tetherin-X1 (*p* < 0.05). These results indicate that canine tetherin-X1 has a stronger antiviral function than canine tetherin-X2.

RT–qPCR Analysis

**Figure 6:**
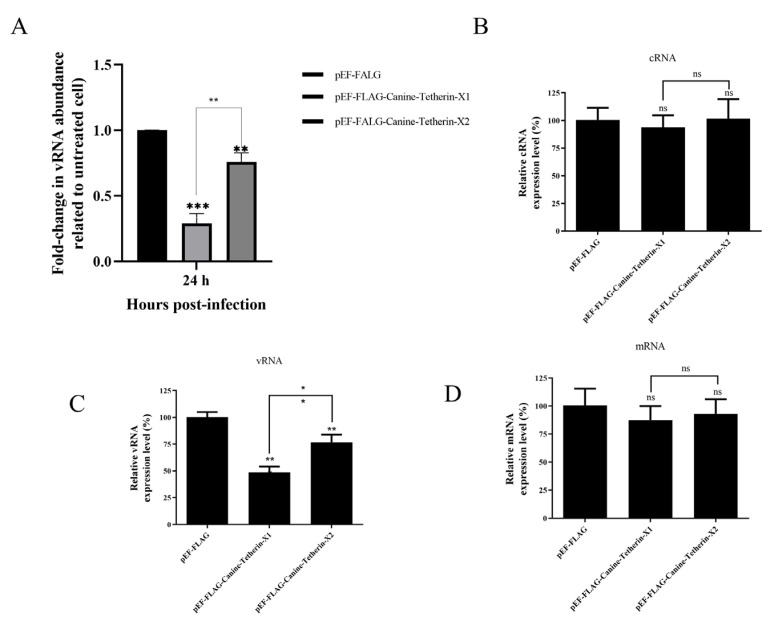
The relative expression levels of CIV RNA were measured with RT–PCR. (**A**) Relative expression levels of vRNA in viral supernatants. (**B**) Relative expression levels of cRNA in infected cells. (**C**) Relative expression levels of vRNA in infected cells. (**D**) Relative expression levels of mRNA in infected cells. Differences in expression were calculated using the 2^−∆∆CT^ method. A *p*-value of < 0.05 was considered to indicate significance (* *p* < 0.05; ** *p* < 0.01; and *** *p* < 0.001); ns indicates a nonsignificant difference.

HEK 293T cells were transiently transfected with the eukaryotic expression plasmids pEF-FLAG, pEF-FLAG-Canine-Tetherin-X1, and pEF-FLAG-Canine-Tetherin-X2. RNA was extracted from the viral supernatants, and cDNA was synthesized via reverse transcription. RT–qPCR was used to measure the relative levels of vRNA in the viral supernatants (Figure 6A). The amount of vRNA in the supernatant of cells transfected with canine tetherin-X1 at 24 hpi was significantly lower than that in the supernatant of empty-vector-transfected cells (*p* < 0.001), as was the amount of vRNA in the supernatant of tetherin-X2-transfected cells (*p* < 0.01). Moreover, the level of vRNA in the supernatant of tetherin-X1-transfected cells was significantly lower than that in the supernatant of tetherin-X2-transfected cells (*p* < 0.01). The relative levels of CIV cRNA, vRNA, and mRNA in infected cells were measured with RT–qPCR, and no significant difference in the relative expression levels of CIV cRNA were found among cells transfected with pEF-FLAG, pEF-FLAG-Canine-Tetherin-X1, and pEF-FLAG-Canine-Tetherin-X2 (Figure 6B). The amount of vRNA in the infected cells transfected with canine tetherin-X1 at 24 hpi was significantly lower than that in the empty-vector-transfected cells (*p* < 0.01), as was the amount of vRNA in the tetherin-X2-transfected cells (*p* < 0.01). Moreover, the level of vRNA in the tetherin-X1-transfected cells was significantly lower than that in the tetherin-X2-transfected cells (*p* < 0.01) (Figure 6C). Moreover, the relative expression level of CIV mRNA in transfected HEK 293T cells did not change significantly after infection with H3N2 CIV (GD/2014) (Figure 6D). These results suggest that tetherin-X1 and tetherin-X2 can restrict the release of nascent H3N2 CIV virions from infected cells.

Western blot Analysis

**Figure 7:**
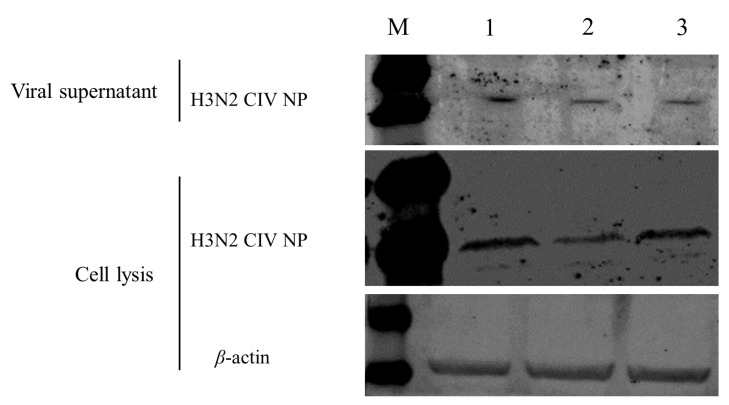
Western blot analysis of transfected HEK 293T cells infected with CIV. Lane M, protein marker; lane 1, pEF-FLAG; lane 2, pEF-FLAG-Canine-Tetherin-X1; and lane 3, pEF-FLAG-Canine-Tetherin-X2.

HEK 293T cells were transfected with pEF-FLAG, pEF-FLAG-Canine-Tetherin-X1, and pEF-FLAG-Canine-Tetherin-X2 and were infected with H3N2 CIV (GD/2014) 24 h after transfection. Proteins were extracted from the viral supernatant and cells 24 h post-infection and analyzed via Western blotting (Figure 7). In the viral supernatant, the band corresponding to H3N2 CIV NP in the empty vector-transfected group was significantly more intense than those in the groups transfected with Tetherin-X1 and Tetherin-X2. In cell lysates, the bands corresponding to H3N2 CIV NP in the groups transfected with tetherin-X1 and tetherin-X2 were less intense than that in the empty vector-transfected group. Moreover, the band corresponding to NP in the tetherin-X1-transfected group was less intense than that in the tetherin-X2-transfected group.

Analysis of the above results indicates that tetherin-X2 can restrict H3N2 CIV release to a certain extent, which is consistent with the activity of tetherin-X1. However, there was a significant difference in the effects of tetherin-X2 and tetherin-X1 on H3N2 CIV restriction. These results indicate that the C-terminus of the coiled-coiled ectodomain and the N-terminus of the GPI-anchor domain of canine tetherin are critical for its ability to restrict H3N2 CIV.

### 3.4. Incidence of Truncated Canine Tetherin Isoforms

**Figure 8:**
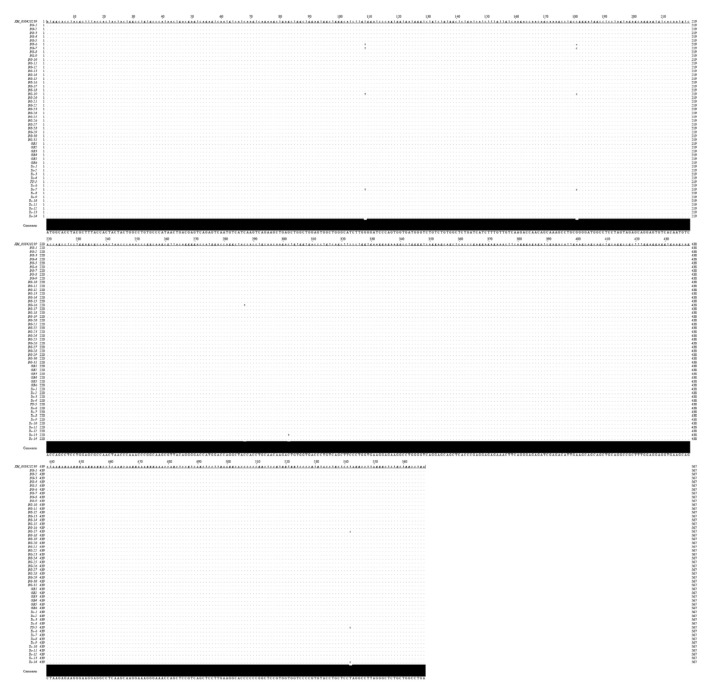
Alignment of the nucleotide sequences of amplified tetherin. Fifty-one canine tetherin genes were amplified from the peripheral blood of different dogs. The canine tetherin sequence (XM_038428239) was downloaded from GenBank. BG indicates that the tetherin gene that was amplified was from a beagle. Tu indicates that the tetherin gene that was amplified was from a mixed-breed dog. GB indicates that the tetherin gene that was amplified was from a poodle. The nucleotide sequences of tetherin were visualized using Jalview software (https://www.jalview.org (accessed on 30 November 2022)).

The incidence of the truncated tetherin isoform in domestic dogs is unclear. Therefore, we collected peripheral blood from beagles, poodles, and mixed-breed dogs and successfully amplified 51 tetherin genes. The nucleotide sequences of the amplified tetherin genes were determined via sequencing. No truncated canine tetherin isoforms were found via nucleotide sequence analysis, and all the obtained tetherin genes had the same number of nucleotides as full-length tetherin (Figure 8). The incidence of truncated tetherin isoforms in canines was 1.96% (1/51).

## 4. Discussion

**Figure 9:**
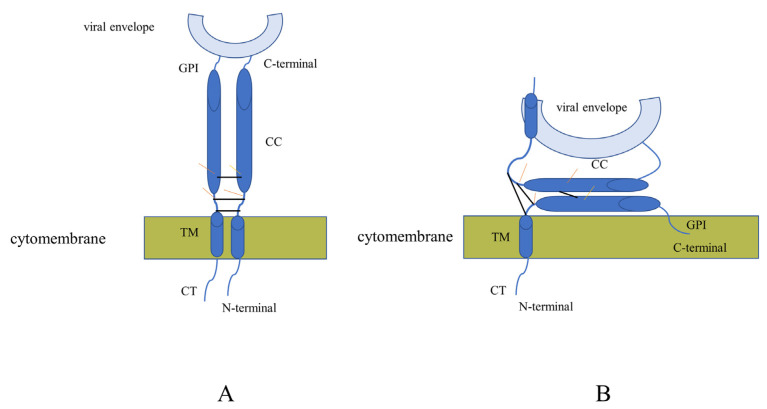
(**A**,**B**) are the hypothesized models of tetherin that restrict enveloped virus release. The black line represents the cysteine site in tetherin. The yellow line represents the glycosylation site in tetherin.

The innate immune system is an important barrier for host cells to resist invasion by external viruses, and tetherin, as an innate immune factor, also plays an important role in resistance to viral invasion [5,24]. Tetherin, an interferon-induced transmembrane protein, was first found to be able to restrict the release of HIV-1 from cells and has since been found to restrict the release of a range of enveloped viruses [7,8]. Tetherin has a unique topology that allows it to form homodimers that enable it to tether nascent progeny virions to the cell membrane surface [25], and we hypothesize two models of tetherin that restrict enveloped virus release (Figure 9). As previously reported, overexpression of human tetherin in MDCK or A549 cells can restrict infection with wild-type influenza viruses or reverse the effects of genetic transfection by influenza viruses [26].

In our study, we amplified canine tetherin from isolated canine peripheral blood leukocytes and identified two isoforms of canine tetherin, tetherin-X1 and tetherin-X2. The sequence analysis showed that tetherin-X2 had a deletion of 34 aa, i.e., aa 147–180, at the C-terminus of the coiled-coiled ectodomain of tetherin and the N-terminus of the GPI-anchor domain. The alignment of nucleotides and amino acids between tetherin-X1 and tetherin-X2 showed that there were mutations at nucleotide 26, 440, and 442 in tetherin-X2, but only at amino acid 9 (Y9C), and the corresponding amino acid sequences at nucleotides 440 and 442 were not changed. Fifty-one canine tetherin genes were amplified from canine peripheral blood, but only one truncated canine tetherin isoform was found. The incidence of the truncated canine tetherin isoform was 1.96%. From the obtained alignment results of 51 canine tetherin sequences, it is interesting that the nucleotide sequence of tetherin in different dogs is still different, and the mutation of this nucleotide sequence does not necessarily lead to amino acid mutation. Although tetherin-X2 has an amino acid deletion, this deletion does not change its subcellular localization; tetherin-X2 is still distributed in the cell membrane, which is consistent with the cellular localization of full-length canine tetherin-X1. Studies have shown that deletion of the coiled-coiled ectodomain or GPI domain of human tetherin results in low expression of human tetherin and loss of or a reduction in viral restriction. Transfection with the same amounts of the tetherin-X2 and tetherin-X1 plasmids resulted in a slightly lower expression of tetherin-X2 than of tetherin-X1. This effect may be due to the partial deletion of the CC domain and GPI domain of tetherin-X2, which results in decreased expression of tetherin-X2. Prediction of the 3D structure of tetherin-X2 shows that the tetherin-X2 spatial structure is consistent with that of tetherin-X1, suggesting that the loss of these amino acids did not lead to a change in the structure of tetherin-X2.

The overexpression of canine tetherin-X1 in HEK 293T cells can restrict the release of H3N2 CIV. Similarly, tetherin-X2 restricts the release of H3N2 CIV. Importantly, the viral titers in cells transfected with tetherin-X2 were higher than those in cells transfected with tetherin-X1. The results of the relative expression level of CIV vRNA in the viral supernatant were consistent with that measured via the TCID_50_. Moreover, the relative expression levels of CIV cRNA and mRNA in the infected cells were not significantly different among the groups. This indicates that tetherin-X1 and tetherin-X2 may be responsible for confining H3N2 CIV to the cell membrane surface. However, the vRNA expression levels were significantly lower in both the tetherin-X1- and tetherin-X2-transfected groups than in the control group. This may be due to the stimulation of the expression of tetherin by the IFN in the host cells after CIV infection, which has a certain inhibitory effect on the replication of the nascent virions, resulting in a decrease in the relative expression level of CIV vRNA. The difference in CIV vRNA between tetherin-X1 and tetherin-X2 also further illustrates the importance of the coiled-coiled ectodomain and GPI domain of canine tetherin for restricting the release of CIV. Based on the analysis of the above results, we hypothesized that model B might be a better explanation for the mechanism of canine tetherin in restricting CIV release (Figure 9B).

Previous studies have shown that deletion of the CT domain, coiled-coiled ectodomain, or GPI domain of human tetherin can abolish its ability to restrict the release of HIV-1 [27]. Moreover, simultaneous mutation of both N-glycosylation sites in tetherin abolished its antiviral activity [27]. To date, few studies have been conducted on the key structural domains or key sites mediating the restrictive effect of canine tetherin on H3N2 CIV release, and extensive research is required to clarify the definite role of tetherin in the spread of viruses between cells. Understanding the mechanism by which tetherin restricts CIV replication and budding and the domains through which tetherin plays a key role will provide insights into virus–host cell interactions and reveal targets for antiviral therapy.

## Data Availability

The data that support the findings of this study are available from the corresponding author upon reasonable request.
